# Thoughts on the Origin of Moles (Germen Falsum), and on Certain Properties of the Secale Cornutum, Illustrated by Cases

**Published:** 1849-04

**Authors:** Thomas Lipscomb

**Affiliations:** Shelbyville, Tenn.


### WESTERN JOURNAL

OF

### MEDICINE AND SURGERY.

APRIL, 1849.

ART. I.—*Thoughts on the Origin of Moles (Germen Falsum), and on certain properties of the Secale Cornutum, illustrated by Cases.* By Dr. THOMAS LIPSCOMB, of Shelbyville, Tenn.

I am not sure that I shall be able to offer any new thoughts on the subjects embraced in the above caption, but having had my attention a good deal directed to them, and having collected a number of facts which, in a practical point of view, appear to me valuable, I have determined to submit the results of my observations to the profession. As to the origin of Moles it is known that there is still uncertainty. Generally they are regarded as imperfect conceptions, but then there are well authenticated instances of moles found in the virgin. Most writers concur in the belief that coition is indispensable to their production, and my own observation has not furnished me with any reasons for dissenting from this prevalent opinion, but the fact just alluded to that they have been met with in undoubted virgins shows that the current doctrine on the subject is not well founded.

My object on the present occasion is to call the attention of the profession to the very frequent occurrence of moles in connection with *protracted lactation.* Can it be a mere coincidence of circumstances? I think not. It is freely admitted that we sometimes meet with them under other and different circumstances, yet if my observation is correct, a very large majority of the cases occur in connection with, and as I believe, under the influence of unnecessarily prolonged nursing. To illustrate my precise meaning: A woman gives birth to a child which she nurses for ten or twelve months, and during this period the secretion of milk exerts so potent an influence on the uterus that the catamenia does not appear. But, if I may be allowed the expression, the uterus refuses to remain dormant any longer, and the menstrual flux once more occurs with its wonted periodicity.—The nursing is continued, and often continued for no other purpose than the prevention of conception; but the object is in part frustrated, for under these circumstances conception does frequently take place. And I may ask how very often does conception under such circumstances prove an imperfect, blighted, or false conception? Might we not anticipate such a result, for although it may still be alleged that conception does not take place in the uterus, yet all perhaps agree that a vigorous and healthy condition of that organ is generally a necessary prerequisite to the development of a healthy fetus. And I think this necessity is proved to be more apparent since the publication of Tracts on generation by Professor Bischoff, of Giessen, by whose experiments the old doctrine of ovarian conception is seriously invalidated, if not positively overthrown. You are doubtless aware that his experiments led him irresistibly to the conclusion that the mature ovum is discharged into the uterus at every menstrual period, and during its stay in that cavity is fecundated by the seminal fluid of the male. The strong and intimate sympathetic influence which continually plays between the uterus and the mammary glands, and *vice versa*, is well known. How frequently do we discover a deterioration of the mother's milk by its effects upon the health of her offspring, simultaneously with the recurrence of the catamenia! We have all witnessed this.

In a period of eighteen years, during which time I have been engaged in the practice of medicine, quite a considerable number of cases of mole, or false conception have fallen under my observation; and without hesitation I assert that a large majority of the whole number have occurred in females who continued to nurse their children after the reëstablishment of the catamenia, and in whom this function was not reëstablished for ten or twelve months after the birth of their children. I have recently solicited the opinion of several of my medical friends whose opportunities for observing have been quite ample. Some of them are satisfied of the correctness of my opinion; others have not paid sufficient attention to the attendant circumstances of their cases to speak definitely on the subject.

Not wishing to be unnecessarily tedious, I will only give a few years from the history of one individual in support of the views above advanced.

During the month of October, 1843, I was consulted by Mrs. ——, who informed me that she had taken cold, which had produced an obstruction of the menstrual discharge some two months previously. She was nursing a child some fifteen months old; and as I am intimately acquainted with her history, I know that she never menstruates until her children are about twelve months old.

This discharge had appeared some three months before, and then ceased to appear at the nsual time. She expressed herself satisfied that pregnancy did not exist, for although a good deal indisposed, she did not suffer the ordinary amount of morning sickness, and experienced periodical pains in the back and lower portion of the abdomen every four weeks. She was much annoyed with a cough of a really serious character, as I thought, threatening phthisis pulmonalis. I urged this as a reason why she should wean her child, hoping if she was not really pregnant, that ceasing to nurse might help to reëstablish the menses, and that she would soon become pregnant, which would aid in suspending the irritation of her lungs. In the course of a few days she was attacked with profuse uterine hemorrhage, when I was sent for. Upon my arrival, I suggested the probability of her laboring under a false conception, and proposed a vaginal examination, which was assented to; I found the os uteri sufficiently patulous to receive the end of my finger, with which I soon detached an organized, fleshy mass, which would probably have weighed between one and two ounces. She now consented to wean her child, and, under appropriate treatment, the irritation of the lungs soon subsided. Soon after regaining her health she conceived again, and in September, 1844, I delivered her of a healthy child, which was probably nursed something over a year. In March, 1847, she gave birth to another healthy child, which she continued to nurse for sixteen or seventeen months, and, as usual, four or five months after the reappearance of the menses. At this time, she was attacked with free hemorrhage from the uterus, when I was requested to visit her, and was informed that there had been no appearance of the catamenia for some two months. I suggested to her the probable existence of another mole, and requested permission to make the necessary examination, to which she very reluctantly assented. I again found the os tincæ patulous, but not being able to feel any substance within with my finger, I availed myself of the wire crotchet, and soon delivered her of a small fleshy mass, after which the hemorrhage ceased. She now, acting under my advice, weaned her child, and is at this time advancing in an apparently healthy pregnancy. This lady is now about thirty-four or thirty-five years of age, has always large and healthy children, enjoys good health herself, is possessed of more than the usual amount of vivacity, and is inclined somewhat to corpulency, her temperament a compound of the sanguine and nervous.

It may very properly be said that one or two cases are insufficient to establish any principle: but many more might be adduced if I did not fear that their history would prove tedious and uninteresting. While engaged in writing this paper, the following case occurred:

February 6th, I was called on to visit Mrs. M., whom I found suffering with pains which she described as being similar to after-pains, attended with very considerable hemorrhage from the uterus. She thought she had taken cold; had not seen any monthly discharge for more than three months. I proposed making a vaginal examination, and in doing so, found the os uteri patulous, and a fleshy mass within, which, with the aid of the wire crotchet, I soon removed, it being a false conception, or mole, about the size of a turkey egg. I delivered this lady of a child about eighteen months previously, which, upon inquiry, she informed me she had nursed steadily until a month since; I made inquiry also as to how long after the birth of her child it was before the catamenia appeared; she said thirteen months. It will be observed that the false conception occurred in this instance under the precise circumstances in which it ought to have taken place upon the theory I am advocating. I will not multiply cases, having given enough to draw attention to the subject.

I proposed in the commencement of these remarks to say a little in reference to the properties of the secale cornutum, or inquire what influences it is capable of exerting on the uterus. Does it merely increase the force of the muscular fibres of the uterus, and promote its tonic contractility, thereby enabling it to contract more perfectly and expel its contents more forcibly? These properties of the ergot are generally admitted, and perhaps sufficiently understood. But are there not other practical indications which may be more successfully fulfilled by it, than by any other therapeutic agent? I am well satisfied there are, and for the confirmation of this opinion I must detail a few years of the history of one individual. Nor do I concieve that the opinion is sustained by this case alone, but derives considerable corroboration from the known and acknowledged properties of the article in question.

Case—Mrs. C., aged about 35 years, had borne five children, the youngest, two and a half years before it walked, from apparent weakness of the spine, of which it was soon relieved by vesicating the whole spinal column, and daily use of the shower bath. Mrs. C. was of lax fibre, quite lean, and a pretty good specimen of the lymphatic temperament. At the period of my being consulted she was suffering with a sanguineous discharge from the uterus, which had existed for some time; thought herself between two and three months advanced in pregnancy. Having been called upon for the relief of the hemorrhage, I recommended rest, acetate of lead, ipecac. and opium, and subsequently, tinct. cinnamon, with tinct. mur. iron, sulphuric acid, counter-irritants to the lumbar region, ext. rhatany, sul. alumna, ext. catechu, etc., etc., with no better effect, than partial abatement and frequent returns of the hemorrhage. This course was persisted in for some two months, by which time the general health was so seriously impaired, and the debility so extreme, that it was evident nothing but a speedy expulsion of the uterine contents could rescue her from impending death. Quickening had already occurred, and although it was perfectly plain that nothing else could avail to save the life of the patient, I thought it best to have the advice of a professional brother, before resorting to means involving so much responsibility. At my suggestion, my friend, Dr. Barksdale, was called in consultation, who immediately concurred with me in opinion as to the necessity of inducing premature labor, as the only hopeful expedient. The expulsive powers of the uterus were soon called into exercise by the secale cornutum, and delivery soon effected, when the hemorrhage immediately ceased. By the free administration of stimulants, external and internal, succeeded by a lengthy and varied course of tonics, Mrs. C. was sufficiently restored to enable her to sit up part of each day and walk through her room occasionally, in the course of three or four months, by which time she conceived again. In about two months thereafter, hemorrhage from the uterus ensued. I was again consulted on account of it. I visited her as frequently as appeared necessary, and again prescribed astringents, anodynes, rest, and tonics, vegetable and mineral, embracing some indigenous plants, but with no better success than before. There was this difference, however, before she was quite as much exhausted as on the previous occasion, the expulsive powers of the uterus were excited, and she was again delivered of a fetus, about the same period of gestation having arrived as when premature labor was induced on the former occasion. On the expulsion of the placenta the hemorrhage ceased. This substance was carefully examined in each case, especially the maternal surface, which very satisfactorily, and I may say conclusively, demonstrated that the separation had been gradually progressing, and that a considerable time had elapsed from the commencement until the separation of the other part; and in each case there was satisfactory evidence that about half of the maternal surface of the placenta had become detached from the uterus previous to delivery. Knowing that more perfect rest could not be attained in any case; that it had been rigidly observed by Mrs. C., as well as the most careful avoidance of every imprudence, I could not but refer the gradual separation of the placenta to the extreme feebleness and delicacy of the connecting medium. My past experience with her afforded me no encouragement to anticipate any benefit in this particular from the use of tonics addressed to the general system. It is true that the extremely feeble state in which this second premature labor left her, called imperiously for the use of general tonics, and they were used as freely, as her stomach would bear in every variety of combination that ingenuity could suggest. And after much reflection upon the case, I determined to add to general tonics, the moderate though perserving use of the secale cornutum, with the hope and design of imparting additional tone and vigor to the uterus.—Months of perseverance in this course, including the use of the shower bath, effected no more, apparently, than the same general remedies had before accomplished. As on the former occasion she acquired only strength enough to enable her to sit up an hour or two each day, and occasionally walk across her room. I feared the occurrence of conception again, to which there seemed an unusual proclivity, notwithstanding the general debility. The event which I deprecated so much again took place in the latter part of 1845, and in February, 1846, she was attacked with uterine hemorrhage. As she was fully aware of her true condition, she soon advised me of it. I determined now at once, that I would not waste important time by the administration of the remedies ordinarily relied upon in such cases, and which had been so fully and unavailingly tried with her. Feeling assured that nothing would avail in her case, except by imparting tone and additional energy to the uterus, I at once commenced with ʒss of the wine of ergot, three times a day, and gradually increased the dose to nearly double that quantity. This plan was persevered in for some two months, with general tonics for the general system. The effect was most happy: the uterine hemorrhage in a few days began to abate, and in the course of two weeks ceased entirely. As above observed, the ergot was persevered in for two months, fearing the hemorrhage might return; but it did not; she continued to enjoy tolerable health. On the last of May she was attacked with diarrhea, accompanied with some tormina. I was sent for, and saw her in the afternoon. I prescribed blue pills, ipecac., and opium, which soon quieted the irritation of the bowels. She rested well through the night, but on the evening of the next day, when the bowels commenced acting again but moderately, the fetal membranes gave way, and the liquor amnii was discharged. I was again summoned to attend her; there being now no chance to prevent premature labor, I exhibited the secale cornutum in sufficient doses to excite expulsive contractions of the uterus, which soon resulted in the expulsion of a male fetus, pretty fully developed for that period of gestation. I now made an examination, and found that there was another fetus still enclosed in its appropriate membranes, which I found upon a subsequent examination and attempt to rupture, unusually dense and strong, as, I do not doubt, the membranes of the other fetus had been as unusually weak. The last fetus expelled was a male also and as large as its fellow.

There was no hemorrhage on this occasion; and, as I think, but for the extreme delicacy of the membranes of the first fetus, Mrs. C. would have reached in safety the ordinary termination of utero-gestation. That this very desirable result would have been attained mainly, if not entirely, through the agency of the ergot, I think none can doubt. It accomplished all for which it was administered; it prevented the detachment of the placenta in this instance, which had occurred on two former occasions; and thereby speedily and effectually controlled the hemorrhage which had commenced as before, and at just about the same period of utero-gestation. This, too, was accomplished under somewhat less favorable circumstances than before existed; for all will admit the powerful influence of habit, even in the production and maintenance of diseased action; and this was really the fourth time that hemorrhage had occurred under the same circumstances with this lady. The first time was previous to my acquaintance with her, and of course I could not detail the particulars of it, but the period of occurrence, and the result were the same as in the two first instances of which I have given a detailed account. Unfortunately, the husband of Mrs. C. died in about two months after her last labor, thereby preventing any farther opportunity of testing the remedy with her, and I have not met with a similar case since.

Professor Dewees recommended the ergot in cases of menorrhagia of long continuance, where the continuance of the discharge was more annoying than the quantity injurious, but not in cases where pregnancy existed. It will be observed that I used it for a different purpose, that of imparting specific tone and energy to the uterus, and that an early period of pregnancy, for the purpose of maturing a firmer connection between the placenta and uterus, and thereby remedying that peculiar state of things to which the hemorrhage owed its existence.

There are other practical indications which can be equally if not more successfully accomplished by ergot than by any other medicinal agent. I will only mention one, and for this purpose I do not recollect to have seen it recommended, although readily inferable from its acknowledged properties. I mean for the prevention or cure of after-pains.

February, 1849.

